# Impact of Coinfection With SARS-CoV-2 and Influenza on Disease Severity: A Systematic Review and Meta-Analysis

**DOI:** 10.3389/fpubh.2021.773130

**Published:** 2021-12-10

**Authors:** Zhou Guan, Can Chen, Yiting Li, Danying Yan, Xiaobao Zhang, Daixi Jiang, Shigui Yang, Lanjuan Li

**Affiliations:** ^1^State Key Laboratory for Diagnosis and Treatment of Infectious Diseases, National Clinical Research Centre for Infectious Diseases, Collaborative Innovation Centre for Diagnosis and Treatment of Infectious Diseases, The First Affiliated Hospital, Zhejiang University School of Medicine, Hangzhou, China; ^2^Women's Hospital, School of Medicine, Zhejiang University, Hangzhou, China

**Keywords:** SARS-CoV-2, influenza, coinfection, meta-analysis, disease severity

## Abstract

**Background:** Although coinfection with influenza in COVID-19 patients has drawn considerable attention, it is still not completely understood whether simultaneously infected with these two viruses influences disease severity. We therefore aimed to estimate the impact of coinfected with SARS-CoV-2 and influenza on the disease outcomes compared with the single infection of SARS-CoV-2.

**Materials and Methods:** We searched the PubMed, Web of Science, Embase, Cochrane Library, China National Knowledge Infrastructure Database (CNKI) to identify relevant articles up to July 9, 2021. Studies that assessed the effect of SARS-CoV-2 and influenza coinfection on disease outcomes or those with sufficient data to calculate risk factors were included. Risk effects were pooled using fixed or random effects model.

**Results:** We ultimately identified 12 studies with 9,498 patients to evaluate the risk effects of SARS-CoV-2 and influenza coinfection on disease severity. Results indicated that coinfection was not significantly associated with mortality (OR = 0.85, 95%CI: 0.51, 1.43; *p* = 0.55, *I*^2^ = 76.00%). However, mortality was found significantly decreased in the studies from China (OR = 0.51, 95%CI: 0.39, 0.68; *I*^2^ = 26.50%), while significantly increased outside China (OR = 1.56, 95%CI: 1.12, 2.19; *I*^2^ = 1.00%). Moreover, a lower risk for critical outcomes was detected among coinfection patients (OR = 0.64, 95%CI: 0.43, 0.97; *p* = 0.04, *I*^2^ = 0.00%). Additionally, coinfection patients presented different laboratory indexes compared with the single SARS-CoV-2 infection, including lymphocyte counts and APTT.

**Conclusion:** Our study revealed that coinfection with SARS-CoV-2 and influenza had no effect on overall mortality. However, risk for critical outcomes was lower in coinfection patients and different associations were detected in the studies from different regions and specific laboratory indexes. Further studies on influenza strains and the order of infection were warranted. Systematic testing for influenza coinfection in COVID-19 patients and influenza vaccination should be recommended.

## Introduction

In December 2019, an outbreak of virus pneumonia caused by a novel coronavirus with high similarity to severe acute respiratory syndrome coronavirus (SARS-CoV) emerged and was subsequently named as SARS-CoV-2 by the World Health Organization (WHO) (1–3). In 2020, the ongoing pandemic of Coronavirus disease 2019 (COVID-19) has posed a great challenge to health care systems globally and has therefore been recognized as a crucial public health emergency of international concern (4). To date (August 4, 2020), WHO has declared more than 197 million confirmed cases and 4,219,861 deaths worldwide (5).

As SARS-CoV-2 continues to spread globally, it will overlap with influenza virus infection in every coming flu season (6). Influenza virus and SARS-CoV-2 infection share similar respiratory illness symptoms, including fever, couch, dyspnea, sore throat, and fatigue, whereas there lack distinctive symptoms to differentiate COVID-19 from influenza infection. Additionally, both viruses can lead to life-threatening outcomes, involving acute respiratory distress syndrome (ARDS), septic shock, and multiple organ failure, especially in older adults and those with chronic diseases and coinfections (7–9).

Effects of coinfection on influenza pneumonia patients have already been well-focused (10). However, it is still not completely understood whether simultaneously infected with SARS-CoV-2 and influenza virus contributes to increased disease severity, in terms of mortality, incidence of shock, being admitted to an intensive care unit (ICU) or requiring ventilatory support. Additionally, knowledge of pathogenic interactions between SARS-CoV-2 and influenza virus is also limited up to now. Therefore, it is crucial to determine the epidemiological impacts of such interaction at present so as to inform treatment and control strategies to contain coinfection with SARS-CoV-2 and influenza virus.

Since the beginning of COVID-19 pandemic, coinfection with influenza virus in COVID-19 patients has been widely detected and a number of case reports were subsequently published (11, 12). However, there is a propensity of case reports reflecting more severe patients. Observational studies with systematic analysis of clinical outcomes in coinfected patients compared with those mono-infected are limited. The conclusions of existing original studies varied because of their small sample size, different study participants, etc. (13, 14). Considering the magnitude of the ongoing COVID-19 pandemic and the need for effective therapeutics, timely meta-analyses can play an important role in assessing the effects of influenza coinfection among COVID-19 patients on clinical outcomes such as mortality. A previous meta-analyze was conducted to evaluate the rate of influenza coinfection among COVID-19 patients, but failed to assess the effects of coinfection on various disease outcomes (15).

The aim of this systematic review and meta-analysis was to evaluate the impact of coinfection with SARS-CoV-2 and influenza on mortality, critical outcomes involving shock, being admitted to ICU or requiring ventilatory support, as well as relevant clinical symptoms and laboratory index compared with mono-infections of SARS-CoV-2.

## Methods

The present study was a multistage systematic review and meta-analysis on a globe scale, which was conducted according to the instruction of the Preferred Reporting Items for Systematic Reviews and Meta-Analyses (PRISMA) (16).

### Search Strategy

For this systematic review and meta-analysis, a computerized search of published literature was performed in PubMed, Web of Science, Embase, Cochrane Library, China National Knowledge Infrastructure Database (CNKI) to identify all relevant articles from database inception to July 9, 2021. These searches were conducted using a comprehensive set of search terms established in collaboration with a librarian specialist. Details of search strategy in each database are available in Supplementary Table 1. We also hand-searched the reference lists of relevant systematic reviews and retrieved primary studies to find references not identified in the computerized searches. No addition of any limit such as language, species, or article types was placed during the search process. Articles and citations were managed with EndNote (version X9).

### Inclusion and Exclusion Criteria

Studies included in this meta-analysis had to satisfy the following criteria: (1) Study subjects were confirmed COVID-19 patients and received diagnostic testing for influenza virus during their infection of SARS-CoV-2 (detected by PCR or serum antibody). (2) Studies had to include a control group, et.al., confirmed COVID-19 patients without influenza infection simultaneously. (3) Studies included a clear description of the disease outcomes of study objects, et.al, death, shock, being admitted to ICU, requiring ventilatory support, and etc., and contained sufficient original data to calculate risk effects between coinfection and control groups. Studies reporting no coinfections or without a clear description of disease outcomes, narrative reviews, meta-analyses, commentaries, editorials, conference abstract, and *in vitro* and *in vivo* animal studies were excluded.

### Screening and Data Extraction

After removing duplicate, titles, and abstracts of all identified studies were carefully reviewed by two investigators (GZ and CC) independently. Unrelated studies that clearly contained no impact of SARS-CoV-2 and influenza coinfection or no available data for risk factors calculations were discarded. All retained full-text articles that potentially eligible were scrutinized according to the predefined eligibility criteria by two independent reviewers (GZ and CC) for their inclusion. Discrepancies and uncertainties in eligibility were resolved by consensus with a third reviewer (YSG).

Data of eligible studies were extracted into a common data extraction template. For each included study, the following information was extracted: first author name, journal name, publication date, study period, country of study population, study design, demographic information, number of participants, and outcomes in both coinfection and control groups (death, shock, being admitted to ICU, and requiring ventilatory support), relevant clinical symptoms and laboratory index, hospital stay, subtype of influenza virus if available. If there were studies that involved multiple outcomes or index, data were extracted separately into corresponding database in this meta-analysis. Two investigators (GZ and CC) performed data extraction independently, and discrepancies were resolved by consensus.

This systematic review and meta-analysis was registered in the International Prospective Register of Systematic Reviews (PROSPERO) with the registration number of CRD42021267039.

### Quality Assessment

Two reviewers (GZ and CC) assessed the methodological quality of selected studies independently using the Newcastle Ottawa Scale (NOS) for observational studies (17). This scale assesses the quality of studies by three major aspects, et.al., selection of the studies, comparability, and outcome/exposure (17). Quality assessment was conducted using the ROBINS-I tool for non-randomized trials (18). Any discrepancy between two reviewers were resolved by consensus with a third reviewer (YSG).

### Statistical Analysis

A primary meta-analysis was conducted to compare mortality between COVID-19 patients coinfected with influenza and control group of SARS-CoV-2 mono-infection. We also assessed the relationship of other critical outcomes, including shock, being admitted to ICU and requiring ventilatory support, with coinfection. Comparison of relevant clinical symptoms and laboratory index were involved in present study. To evaluate the discrepancy of these indicators between two groups, data from original studies were pooled and expressed as odds ratio (OR) with their 95% confidence intervals (CI) in case of categorical data, while mean difference (MD) with their 95% CI in case of continuous data. For studies that presented continuous data as medians and inter-quartile ranges, the estimate of their means, and standard deviations was carried out based on the statistical formula described by Luo et al. (19). Inter-study heterogeneity was assessed by Cochrane's *Q* and quantified by *I*^2^. If *I*^2^ >50%, a random-effect model was used to calculate the effect value. Otherwise, a fixed-effect model was performed (20, 21). In addition, we used funnel plots to assess the publication bias in this meta-analysis. We also performed Egger's and Begg's test for statistical examination of publication bias (22). Moreover, subgroup effects were evaluated by stratified meta-analysis according to study-level characteristics. Additionally, influence analysis was conducted by serially omitting each study to estimate the effect of individual study on the overall risk estimates (23). All analyses were conducted using R version v3.2.3 (R Foundation for Statistical Computing) and the significant level was set as *P* < 0.05.

## Results

### Literature Search

**Figure 1:**
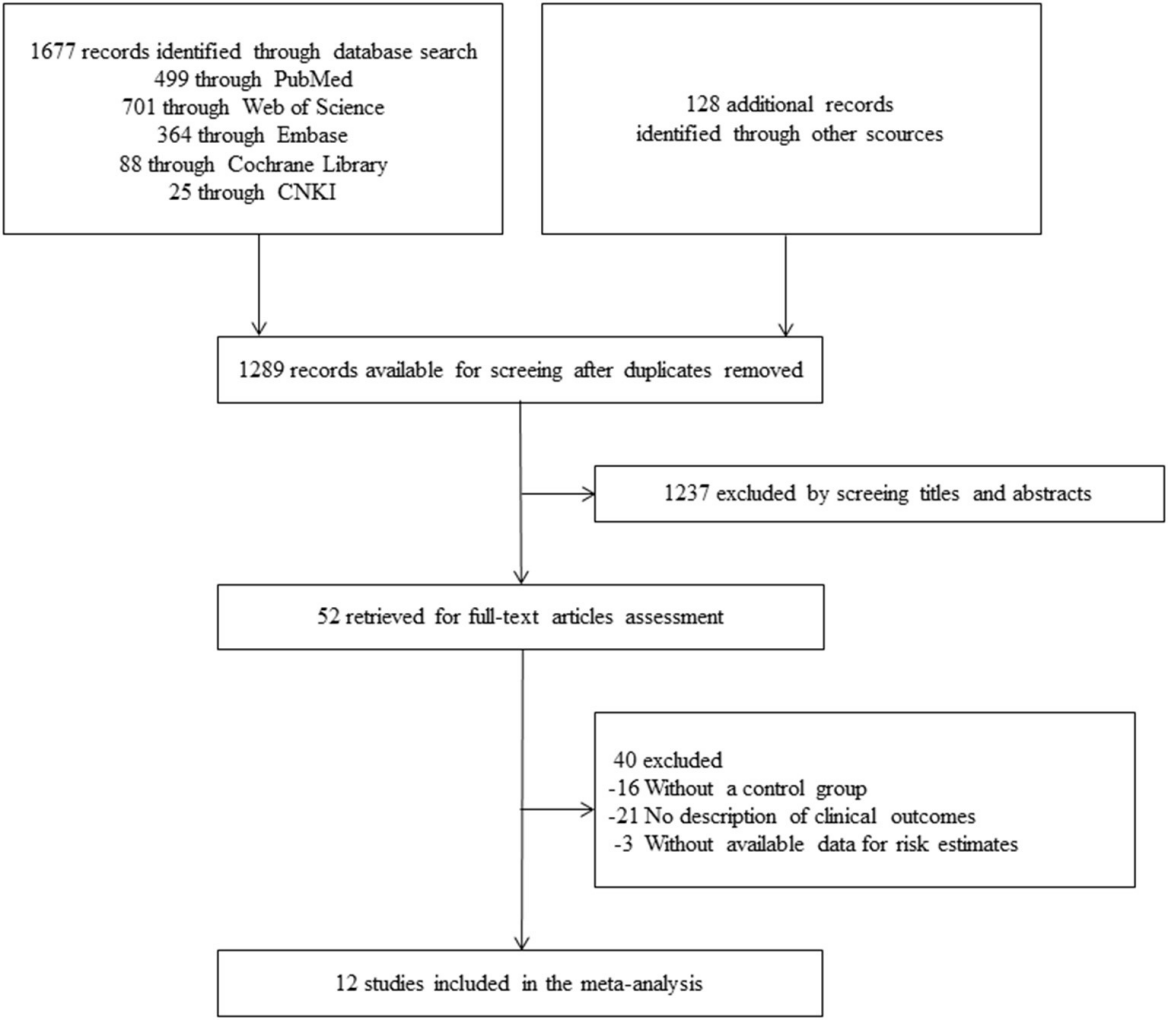
Flowchart of study selection in this systematic review and meta-analysis.

Our literature search identified 1,805 records, among which 1,677 came from database searching and 128 from literature reviews (Figure 1). After removing duplicates, 1,289 records were involved in the initial screening of title and abstract, then 1,237 were excluded according to the pre-specified inclusion and exclusion criteria. In total, 52 potentially relevant full-text articles were independently assessed for eligibility. Among these, 16 records failed to involve a control group in their studies. Twenty-one articles mentioned about coinfection but did not provide data on disease outcomes for both groups. Two studies reported multiple-infections in COVID-19 patients, but the exact number of influenza coinfection were not available (24, 25). One study of veterans failed to provide the total number of COVID-19 patients who simultaneously tested for influenza (26). Therefore, we ultimately identified 12 studies with sufficient data to calculate risk estimation of coinfection with SARS-CoV-2 and influenza (Table 1). Nine of the twelve studies were performed among patients in all age groups, and three were conducted on adult patients. Of the involved studies, a total of 11,674 patients were included, with 1,873 in coinfection group and 9,801 in control group.

**Table 1:** Characteristics of the studies included in this meta-analysis.

**Authors**	**Publishing time**	**Study period**	**Country**	**WHO region**	**Number of coinfection group**	**Number of control group**
Chen et al. (27)	2021.07	2020.01-03	China	Western Pacific	13	348
Tong et al. (28)	2021.04	2020.02-03	China	Western Pacific	10	67
Ma et al. (29)	2020.07	2020.01-02	China	Western Pacific	22	47
Cheng et al. (4)	2021.05	2020.01-03	China	Western Pacific	5	116
Wu et al. (14)	2021.01	2020.01-04	China	Western Pacific	96	660
Hashemi et al. (30)	2021.02	2020.03-04	Iran	Eastern Mediterranean	82	1,163
Stowe et al. (13)	2021.03	2020.01-04	England	Europe	1,193	4,443
Wang et al. (31)	–	2020.02-03	China	Western Pacific	29	122
Yue et al. (32)	2021.01	2020.01-02	China	Western Pacific	6	64
Yu et al. (33)	2021.06	–	Saudi Arabia	Eastern Mediterranean	4	31
Alosaimi et al. (34)	2021.02	2020.03-05	USA	America	222	917
Takahashi et al. (35)	2020.11	2020.01-02	China	Western Pacific	192	141

### Coinfection and Mortality

**Figure 2:**
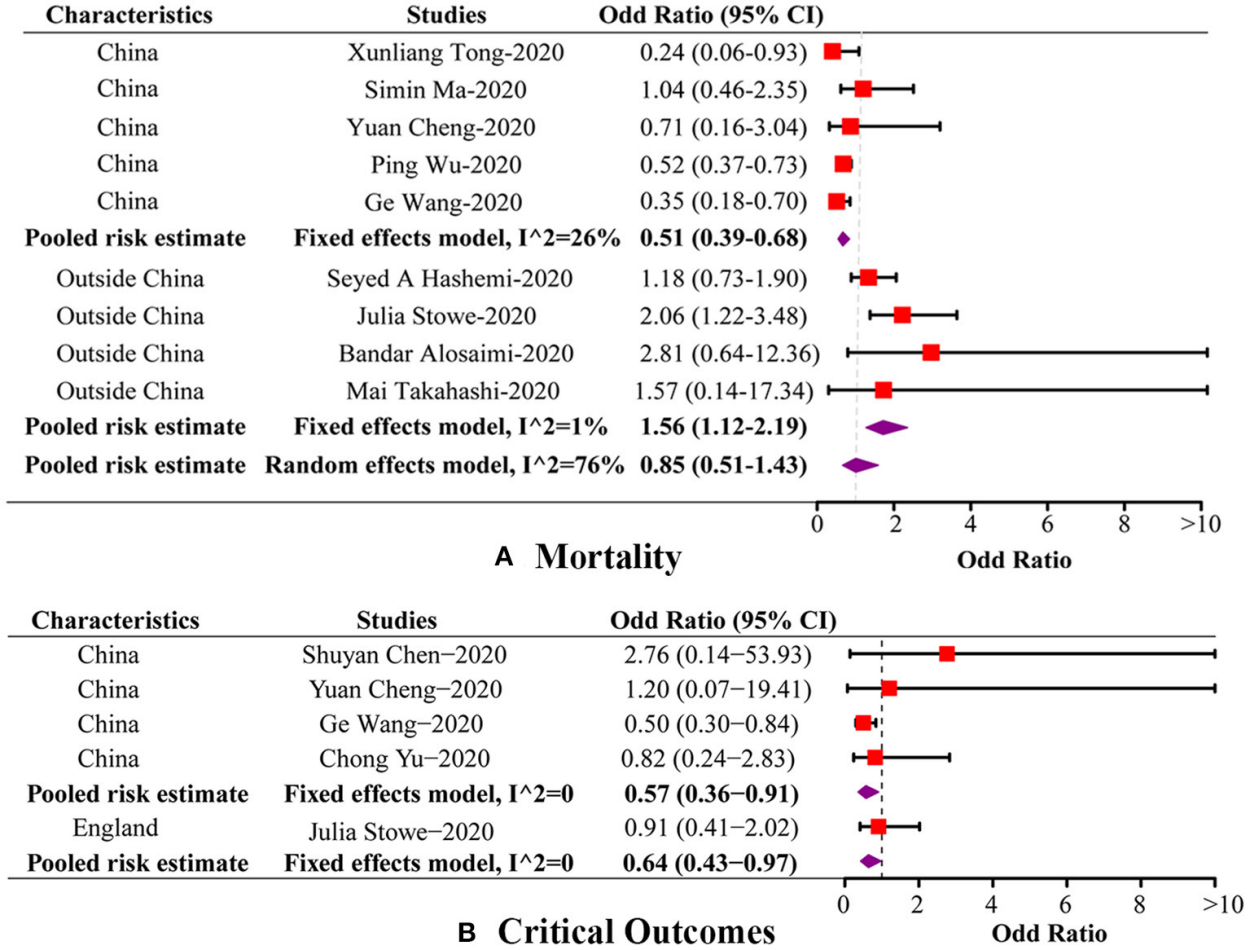
Forest plots of the association between coinfection and disease outcomes by subgroup. **(A)** Forest plot of the association between coinfection and mortality by subgroup. **(B)** Forest plot of the association between coinfection and critical outcomes by subgroup.

**Figure 3:**
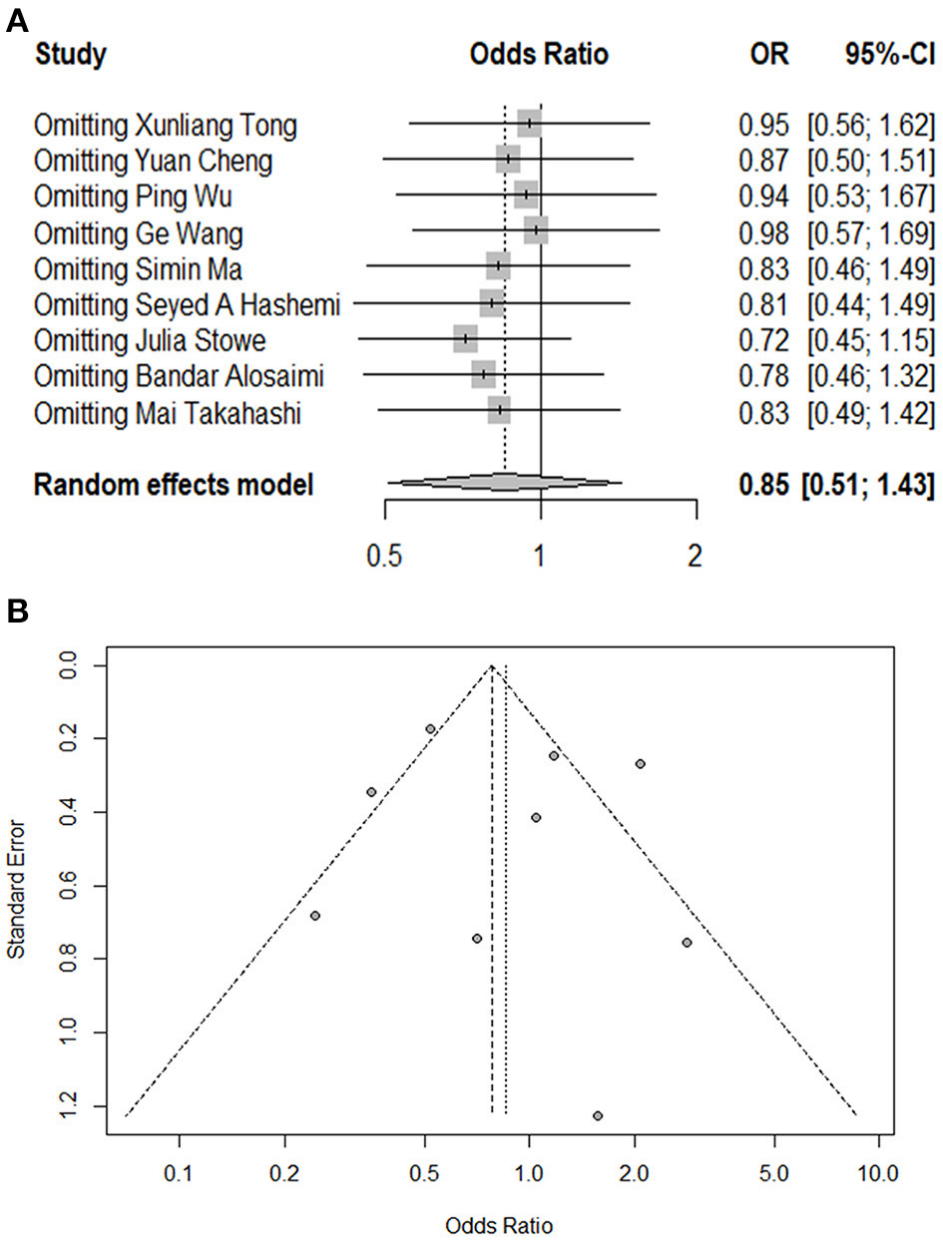
Influential analysis and funnel plot of this meta-analysis. **(A)** Influential analysis of omitting each study on risk estimate. **(B)** Funnel plot assessing publication bias of the included studies.

The present meta-analysis identified nine studies reporting mortality in both groups. The pooled OR for overall mortality was 0.85 (95%CI: 0.51, 1.43; *p* = 0.55, *I*^2^ = 76.00%), which indicated that there was no significant association between coinfection and overall mortality (Figure 2A). In subgroup analysis, there were five studies conducted in China and four outside China, i.e., Iran, England, Saudi Arabia, and USA, respectively. Among the studies from China, mortality was found to be significantly lower in coinfection patients (OR = 0.51, 95%CI: 0.39, 0.68; *I*^2^ = 26.50%). Whereas, in the studies conducted outside China, a significantly increased mortality was detected in coinfection patients (OR = 1.56, 95%CI: 1.12, 2.19; *I*^2^ = 1.00%). Meanwhile, a significant subgroup difference was revealed when we separated studies according to study regions (*P*_*heterogeneitybetween*_ < 0.01). We also divided studies into comparative (*N* = 7) and non-comparative (*N* = 2) study, according to whether their objective was to compare mortality between two groups. However, no significant association was found in both subgroups. Additionally, four of the nine studies involved data on influenza subtypes, namely, influenza A and B, while no significant association was found in both subtypes. We were not able to carry out subgroup analysis of age groups and study periods because of limited data. The results of influential analysis were presented in Figure 3A. The funnel plot indicated that there was a good symmetry around the pooled risk estimates (Figure 3B). Egger's test (*p* = 0.56) and Begg's (*p* = 0.68) test also suggested that there was less likely a publication bias in our meta-analysis.

### Coinfection and Critical Outcomes

In our meta-analysis, there were five studies including sufficient data of critical outcomes, involving shock, being admitted to ICU or requiring ventilatory support. The pooled OR for critical outcomes was 0.64 (95%CI: 0.43, 0.97; *p* = 0.04, *I*^2^ = 0.00%), indicating a decreased risk for critical outcomes in coinfection patients (Figure 2B). Among these five studies, four were conducted in China, and one in England. The pooled OR of the four Chinese studies was 0.57 (95%CI: 0.36, 0.91; *I*^2^ = 0.00%), which was in accordance with the overall risk estimate. One study carried out in England reported no association between coinfection and critical outcomes. Two of the five studies provided data on influenza subtypes, while no significant association was found in both subtypes. In respect of other disease outcomes, two studies used an outcome definition as aggravated or death, and two studies reported data on hospital stay of patients. However, no significant association was detected between coinfection and these outcomes (Table 2).

**Table 2:** Estimated effects of coinfection on disease severity.

**Outcome**	**Number of studies**	**Effect estimate (95%CI)**	**Heterogeneity (*I*^2^)**
**Mortality**	9	OR: 0.85 (0.50–1.43)	76.00%
In China^*^	5	OR: 0.51 (0.39–0.68)	26.50%
Outside China^*^	4	OR: 1.56 (1.12–2.19)	1.00%
Influenza A coinfection	4	OR: 0.73 (0.37–1.46)	80.00%
Influenza B coinfection	2	OR: 0.60 (0.29–1.23)	0.00%
**Critical outcomes** ^*^	5	OR: 0.64 (0.43–0.97)	0.00%
In China^*^	4	OR: 0.57 (0.36–0.91)	0.00%
Outside China	1	OR: 0.91 (0.41–2.02)	–
Influenza A coinfection	3	OR: 1.63 (0.28–9.52)	86.20%
Influenza B coinfection	3	OR: 1.38 (0.63–3.03)	48.40%
**Aggravated or death**	2	OR: 1.13 (0.58–2.19)	0.00%
**Hospital stay**	2	MD: 1.02 (−1.31 to 3.34)	52.90%
**Clinical symptoms**			
Fever	5	OR: 1.02 (0.71–1.45)	26.90%
Dyspnea	5	OR: 0.75 (0.55–1.02)	0.00%
Cough	5	OR: 0.86 (0.64–1.15)	0.00%
**Laboratory index**			
White blood cells	3	MD: 0.08 (−0.37 to 0.53)	46.90%
Lymphocytes^*^	6	MD: 0.07 (0.003–0.13)	0.00%
IL-6	5	MD: −1.12 (−3.58 to 1.34)	22.20%
CRP	3	MD: −7.20 (−16.81 to 2.42)	0.00%
APTT^*^	2	MD: −1.80 (−3.11 to −0.49)	0.00%
IL2R	2	MD: −82.37 (−372.53 to 207.78)	89.20%

*OR, odds ratio; MD, mean deviation; CI, confidence interval*.

* *P < 0.05 significant*.

### Clinical Symptoms and Laboratory Index

In addition to the outcomes described previously, we also extracted data of clinical symptoms and laboratory index of patients (Table 2). Five studies provided sufficient data on clinical symptoms, while no significant association was detected between coinfection and any of the clinical symptoms, such as fever (OR = 1.02, 95%CI: 0.71, 1.45; *p* = 0.93, *I*^2^ = 26.90%), cough (OR = 0.86, 95%CI: 0.64, 1.15; *p* = 0.31, *I*^2^ = 0.00%) and dyspnea (OR = 0.75, 95%CI: 0.55, 1.02; *p* = 0.07, *I*^2^ = 0.00%). In respect of laboratory index, the reported terms varied between studies and a majority of index, such as white blood cells, C-reactive protein, IL-6, and IL2R, showed no significant difference between two groups. However, the lymphocyte counts were found significantly higher in coinfection group (*N* = 6, *MD* = 0.07, 95%CI: 0.003, 0.13; *p* = 0.04, *I*^2^ = 0.00%). Activated partial thromboplastin time (APTT) was found to be significantly longer in control group (*N* = 2, *MD* = −1.80, 95%CI: −3.11, −0.49; *p* = 0.007, *I*^2^ = 0.00%).

## Discussion

Under the background of COVID-19 global pandemic, the number of patients coinfected with SARS-CoV-2 and influenza may increase worldwide in the coming cold seasons. Both viruses can cause critical outcomes and death, especially among vulnerable populations, which therefore draw substantial concern. Generally, coinfection is considered to lead to more severe symptoms and eventually worsen the disease outcomes. A mechanism study indicated that coinfection may modify the virulence of virus, and therefore altering disease severity (36). A mathematical model study to determine the dynamics of viral coinfection also showed that virus species and growth rate may affect the replication of other viruses (37). To date, observational studies have reported three distinctive associations between coinfection and clinical outcomes, i.e., improved, deteriorated, and no effect. These contradictory results imply a complicated mechanism on how coinfection affects mortality and critical outcomes, and it is exactly where further researches are urgently needed.

To our knowledge, this is the first comprehensive meta-analysis conducted to determine the impact of coinfection with SARS-CoV-2 and influenza on mortality, critical outcomes involving shock, being admitted to ICU, and requiring ventilatory support, as well as relevant clinical symptoms and laboratory index. Our results showed that coinfection with influenza virus was not associated with overall mortality in COVID-19 patients. However, in our subgroup analysis, two opposite patterns were revealed. Mortality was found to be significantly decreased in the studies from China. While a significantly increased mortality was detected in the studies conducted outside China. As for critical outcomes, a marginally decreased risk was detected among coinfection patients. Additionally, our results showed that COVID-19 patients coinfected with influenza presented with clinical symptoms and laboratory indexes similar to those with single SARS-CoV-2 infection, except for lymphocyte counts and APTT. Our meta-analysis reported a high heterogeneity for overall mortality, but this heterogeneity was considerably declined among subgroup analysis, which may explain part of the heterogeneity sources.

An interesting observation revealed by our subgroup meta-analysis was that, studies from China showed a significantly decreased risk of mortality compared to those outside China, which might be explained by following points. Firstly, the differences in races and influenza strains among different countries may lead to different clinical outcomes among COVID-19 patients coinfected with influenza. However, our meta-analysis only included limited studies involving influenza strains. At present, some observational study showed that patients positive for influenza A IgM had a lower risk of mortality (14), and some reported that influenza B virus coinfection leads to a higher risk of developing adverse prognosis (30). Secondly, at the initial period of COVID-19 outbreak, thousands of patients who were diagnosed as COVID-19 failed to accept examinations of coinfection pathogens due to the huge task of SARS-CoV-2 rapid tests and the lack of widely available testing methods. And this might lead to selection bias in observational studies to some extent. Thirdly, studies included in present meta-analysis were basically conducted within January to May, 2020. Among these, studies from China were generally conducted before March, including one from January to April. While the study periods of those outside China were basically after March, including one from January to April. Thurs, there might be some differences in detection ranges and influenza strains between studies from different countries.

Virus coinfection is generally considered to be a risk factor for adverse clinical outcomes, which was similar to our results of the studies outside China. This finding suggested a possible synergistic effect between SARS-CoV-2 and influenza virus, which has previously been reported between influenza and other respiratory viruses, e.g., by facilitating virus transmission between cells (38). Additionally, coinfection with influenza virus is reported to enhance neutrophil activation, thereby contributing to an excessive immune response against virus and also leading to a cytokine storm. Cytokine storm could further cause massive infiltration of macrophages and neutrophils, which therefore could induce lung tissue injury and worsen disease outcomes (39). A study on animal model also found an increased disease severity among hamsters coinfected with SARS-CoV-2 and influenza A compared with those with single SARS-CoV-2 infection (40).

Coinciding with some original studies, our subgroup analysis revealed a distinctive pattern of a lower risk for mortality and critical outcomes among coinfection patients, which might be related to following reasons. Firstly, increasing number of literatures has been demonstrating that influenza virus infection may trigger non-neutralizing antibodies responses which also binds to other pathogens, like HIV and Ebola (41, 42). However, this mechanism might rely on whether influenza infection occurred prior to SARS-CoV-2. Our study failed to perform this part of analysis due to the limited data. Secondly, significantly lower odds of critical outcomes and mortality were found in COVID-19 patients who received influenza vaccination before in some studies (43, 44). This might imply a potential competitive mechanism between SARS-CoV-2 and influenza, like competitively bound to the receptors and thereby contributing to reduction or block of SARS-CoV-2 entry into lung cells. It is also possible that influenza vaccination could stimulate short-term non-specific immune response that provides a temporary protection against SARS-CoV-2. Thirdly, a considerable reduction of cytokines among coinfection patients was detected in some studies, indicating that patients with coinfection might have lower degree of hyper-inflammation, and therefore lower risk for adverse outcomes (4).

This study is the first comprehensive meta-analysis focused on the impact of SARS-CoV-2 and influenza coinfection on a wide range of disease outcomes. However, our results should still be interpreted with caution due to several limitations. Firstly, a high level of heterogeneity was noted in the meta-analysis of overall mortality. All studies included were conducted in the year of 2020 and about half of them were performed in China. We therefore used a random effects model to deal with the high heterogeneity. In additional, subgroup analyses also helped to interpret part of the heterogeneity sources. Secondly, when analyzing the association between coinfection and disease outcomes, we failed to adjust confounding factors, such as age, underlying diseases, influenza subtypes and the order of infection, because of limited data. At present, there were very few specialized studies focused on the comparison of disease outcomes between coinfection patients and mono-infections. Among the existing studies, the topic of coinfection is generally embedded within the studies that mainly focused on the characteristics of COVID-19 patients. As a result, our research could only include relatively limited number of studies. Therefore, further specialized comparative study with a well-designed control group is warranted to better inform the effect of coinfection on disease outcomes. Additionally, considering the continuous cocirculation of these two viruses, future investigations are urgently needed to collect information on SARS-CoV-2 subtypes, influenza subtypes, sequence of infection, comorbidity, so as to perform stratified analysis and provide more detailed information. Moreover, mechanism studies on the interaction of SARS-CoV-2 and influenza virus are also warranted to further explore the effect of coinfection and provide timely information on prevention and treatment.

## Conclusion

Our meta-analysis revealed that coinfection with SARS-CoV-2 and influenza had no observable effect on the overall mortality. However, several distinctive associations were detected in subgroup analysis of different regions, critical outcomes and laboratory index. These contradictory results therefore require further well-designed controlled studies that adjusts confounding factors like comorbidity, influenza strains and the order of infection. Additionally, studies on the mechanism of coinfection is also urgently needed. Considering that cocirculation of these two viruses could have a considerable impact on morbidity and mortality, systematic testing for influenza coinfection in COVID-19 patients is necessary, and influenza vaccination should be recommended not only to reduce the risk of coinfection, but also for the potential benefits to immune system.

## Author Contributions

LL, ZG, and CC designed the study. ZG and CC developed and implemented the search protocol. YL, DY, XZ, DJ, ZG, and CC abstracted data, with SY acting as a tie-breaker at all stages. ZG and CC performed the statistical analysis and wrote the manuscript. All authors contributed to the article and approved the submitted version.

## Funding

This study was financially supported by the National Natural Science Foundation of China (Grant Nos. 81672005, U1611264, 81001271, and 81721091) and the Mega-Project of National Science and Technology for the 12th and 13th Five-Year Plan of China (Grant Nos. 2018ZX10715-014-002 and 2014ZX10004008).

## Conflict of Interest

The authors declare that the research was conducted in the absence of any commercial or financial relationships that could be construed as a potential conflict of interest.

## Publisher's Note

All claims expressed in this article are solely those of the authors and do not necessarily represent those of their affiliated organizations, or those of the publisher, the editors and the reviewers. Any product that may be evaluated in this article, or claim that may be made by its manufacturer, is not guaranteed or endorsed by the publisher.
