# Supplementary material for: Impact of Coinfection With SARS-CoV-2 and Influenza on Disease Severity: A Systematic Review and Meta-Analysis
Source: Front Public Health. 2021 Dec 10;9:773130. doi: 10.3389/fpubh.2021.773130 (PMC8703010; doi:10.3389/fpubh.2021.773130)
Supplement: Supplementary file 1 [file Data_Sheet_1.docx]

**Supplementary table 1. Literature search strategy**

**PubMed**

| **#** | **Searches** | **Results** |
| --- | --- | --- |
| **#1** | ((("Influenza, Human"[Mesh]) OR (((influenza) OR (flu)) OR (grippe))) AND (("COVID-19"[Mesh] OR "SARS-CoV-2"[Mesh]) OR ((((((severe acute respiratory syndrome coronavirus 2) OR (COVID-19)) OR (SARS-CoV-2)) OR (coronavirus disease 2019)) OR (2019-nCov)) OR (novel coronavirus)))) AND (("Coinfection"[Mesh]) OR ((((((coinfection) OR (co-infection)) OR (secondary infection)) OR (mixed infection)) OR (concomitant)) OR (dual infection))) | **499** |

**Web of Science**

| **#** | **Searches** | **Results** |
| --- | --- | --- |
| **#1** | (((TS=(influenza, human)) OR TS=(influenza)) OR TS=(flu)) OR TS=(grippe) | [229303](https://www.webofscience.com/wos/alldb/summary/8bab164c-81f6-4ade-8280-5c6ebe4879e4-0051fa1c/relevance/1) |
| **#2** | (((((TS=(severe acute respiratory syndrome coronavirus 2)) OR TS=(COVID-19)) OR TS=(SARS-CoV-2)) OR TS=(coronavirus disease 2019)) OR TS=(2019-nCov)) OR TS=(novel coronavirus) | [208182](https://www.webofscience.com/wos/alldb/summary/6d76de7e-37bb-4be6-b2a9-41c1435d6056-005203fb/relevance/1) |
| **#3** | (((((TS=(coinfection)) OR TS=(co-infection)) OR TS=(secondary infection)) OR TS=(mixed infection)) OR TS=(concomitant)) OR TS=(dual infection) | [700920](https://www.webofscience.com/wos/alldb/summary/63d4f99c-0966-40c2-af46-16497aa2dbda-005207c0/relevance/1) |
| **#4** | **((#1) AND #2) AND #3** | **701** |

**EMBASE**

| **#** | **Searches** | **Results** |
| --- | --- | --- |
| **#1** | 'influenza'/exp OR 'influenza' OR influenza OR flu OR grippe | 191444 |
| **#2** | 'severe acute respiratory syndrome coronavirus 2'/exp OR 'coronavirus disease 2019'/exp OR 'severe acute respiratory syndrome coronavirus 2' OR 'covid 19' OR 'sars cov 2' OR 'coronavirus disease 2019' OR '2019 ncov' OR 'novel coronavirus' | 159364 |
| **#3** | 'coinfection'/exp OR 'secondary infection'/exp OR coinfection OR 'co infection' OR 'secondary infection' OR 'mixed infection' OR concomitant OR 'dual infection' | 333864 |
| **#4** | **#1 and #2 and #3** | **364** |

**Cochrane Library**

| **#** | **Searches** | **Results** |
| --- | --- | --- |
| **#1** | MeSH descriptor: [Influenza, Human] explode all trees OR (influenza, human):ti,ab,kw OR (influenza):ti,ab,kw OR (flu):ti,ab,kw OR (grippe):ti,ab,kw (Word variations have been searched) | 11699 |
| **#2** | MeSH descriptor: [SARS-CoV-2] explode all trees OR MeSH descriptor: [COVID-19] explode all trees OR (severe acute respiratory syndrome coronavirus 2):ti,ab,kw OR (COVID-19):ti,ab,kw OR (SARS-CoV-2):ti,ab,kw OR (coronavirus disease 2019):ti,ab,kw OR (novel coronavirus):ti,ab,kw (Word variations have been searched) | 6203 |
| **#3** | MeSH descriptor: [Coinfection] explode all trees OR (coinfection):ti,ab,kw OR (secondary infection):ti,ab,kw OR (mixed infection):ti,ab,kw OR (concomitant):ti,ab,kw OR (co-infection):ti,ab,kw (Word variations have been searched) OR (co-infection):ti,ab,kw (Word variations have been searched) | 51499 |
| **#4** | **#1 and #2 and #3** | **88** |

**CNKI** (China National Knowledge Infrastructure Database)

| **#** | **Searches** | **Results** |
| --- | --- | --- |
| **#1** | (SU%=influenza OR SU%=flu OR SU%=grippe) AND (SU%=severe acute respiratory syndrome coronavirus 2 OR SU%=COVID-19 OR SU%=SARS-CoV-2 OR SU%=coronavirus disease 2019 OR SU%=2019-nCov OR SU%=novel coronavirus) AND (SU%=coinfection OR SU%=co-infection OR SU%=secondary infection OR SU%=mixed infection OR SU%=concomitant OR SU%=dual infection) | **25** |
